# Doege-Potter syndrome due to a solitary fibrous tumor of the pleura: a case report

**DOI:** 10.1186/s13256-024-04658-1

**Published:** 2024-08-14

**Authors:** Juan Estrada-Maya, Juan Sebastián Montejo, Katerin Dayana Báez López, Juan Carlos Garzón

**Affiliations:** 1 Internal Medicine, Fundación Cardioinfantil-Instituto de Cardiología, Calle 163ª#13B-60, Bogotá, Colombia; 2 School of Medicine and Health Sciences, Universidad del Rosario, Bogotá, Colombia; 3 General Medicine, Fundación Cardioinfantil-Instituto de Cardiología, Bogotá, Colombia; 4 Thoracic Surgery, Fundación Cardioinfantil-Instituto de Cardiología, Bogotá, Colombia

**Keywords:** Solitary fibrous tumor, Hypoglycemia, Insulin-like growth factor II, Doege-Potter syndrome, Paraneoplastic syndrome

## Abstract

**Background:**

Doege-Potter syndrome is a rare paraneoplastic phenomenon associated with solitary fibrous tumors of the pleura (SFTPs). It is characterized by the presence of severe, sustained, and treatment-refractory hypoglycemia. Hypoglycaemia, which may be the sole symptom at disease onset, is mediated by the secretion of high-molecular-weight insulin-like growth factor (IGF-2). Most tumors exhibit benign behavior, with a 100% survival rate at 5 years. However, 10% of these tumors may display aggressive behavior with local or metastatic recurrence. We present a clinical case of a patient with a benign solitary fibrous tumor of the pleura who presented with symptomatic hypoglycemia and required pulmonary and pleural surgical resection to control the paraneoplastic phenomenon.

**Case presentation:**

A Hispanic 46-year-old man presented with a 15-day history of transient alterations in consciousness worsened by fasting. The relevant medical history included obstructive sleep apnea treated with continuous positive air pressure (CPAP) and previous smoking. In-hospital studies revealed noninsulinemic hypoglycemia and a benign SFTP. Complete surgical resection was performed while the patient received dextrose fluids and corticosteroids perioperatively for hypoglycemia. Subsequently, the hypoglycemia resolved, and the patient was followed-up without disease recurrence.

**Conclusion:**

Doege-Potter syndrome is challenging to recognize. However, effective treatment can be achieved with a high survival rate. Raising awareness among healthcare professionals about the recognition of this paraneoplasic syndrome patients will improve diagnostic suspicion, biochemical confirmation, the development of diagnostic and therapeutic guidelines, and the creation of predictive indices for aggressive presentations requiring closer monitoring.

## Background

Non-Islet Cell Tumor Hypoglycemia (NICTH) is an uncommon paraneoplastic condition caused by the synthesis of insulin-like growth factor type 2 (IGF-2) by benign and malignant nonpancreatic tumors [1]. This condition, first described by Karl Doege and Roy Potter in 1930 is characterized by recurrent and refractory hypoglycemia in the presence of an intrathoracic solitary fibrous tumor [2]. Doege-Potter syndrome is attributed to non-insulin-induced hypoglycemia caused on by fibrous tumors, while it can also be seen in mesotheliomas, liposarcoma, rhabdomyosarcoma, leukemia, lymphoma, and teratoma [3].

Solitary fibrous tumors are mesenchymal neoplasms that make up 2% of soft tissue tumors [4]. While they typically develop in the pleura, they can also affect the peritoneum, pericardium, and mediastinum [5]. Although rare, they occur at a rate of 2.8 per 100,000 people and usually have an indolent and benign course in 90% of cases [6]. However, about 5% of patients develop difficult-to-manage non-insulin-induced hypoglycemia [7, 8].

Doege-Potter syndrome patients are difficult to identify since their condition is rarely taken into account while making a differential diagnosis of hypoglycemia. Case reports and short series make up the majority of the literature on Doege-Potter syndrome [9–16]. By sharing our experience and highlighting interesting aspects of the pathophysiology, our case report seeks to advance understanding of this syndrome.

## Case presentation

We present the case of a Hispanic 46-year-old male from Bogotá, Colombia, with a 15-day history of disorientation, altered thought content, stereotyped movements of the upper limbs, and episodes of heteroaggressive behavior. The patient, who was diagnosed with obstructive sleep apnea (OSA) using continuous positive air pressure (CPAP), had a history of smoking cessation (5 pack-years) and a recently identified suspicious right lung mass on outpatient chest computed tomography (CT). There was no other relevant personal or family history.

**Fig. 1:**
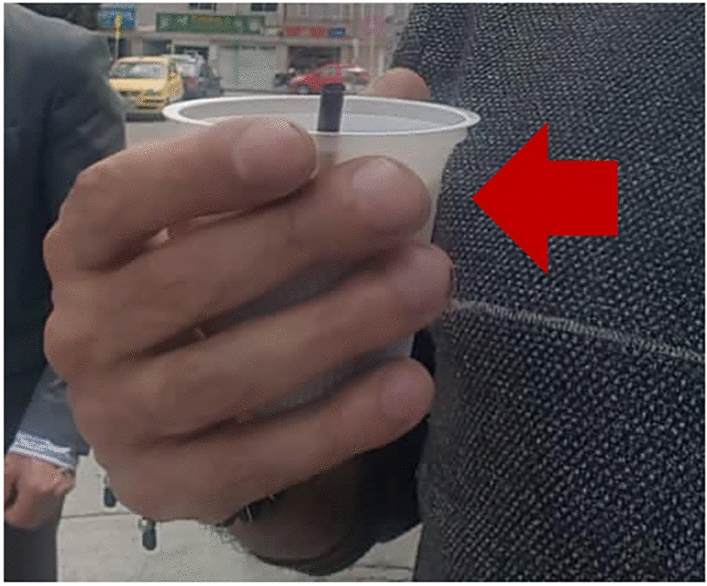
Image of the patient highlighting physical examination findings: digital clubbing indicated by a red arrow. The patient's face has been obscured to protect his identity

Upon admission to the CardioInfantil Foundation-Cardiology Institute, the patient was in good condition with all vital signs at normal ranges. Physical examination revealed digital clubbing (Fig. 1) in both hands, skin thickening between the eyes, also known as pachydermoperiostosis, absence of breath sounds in the right hemithorax, and a normal neurological examination.

**Fig. 2:**
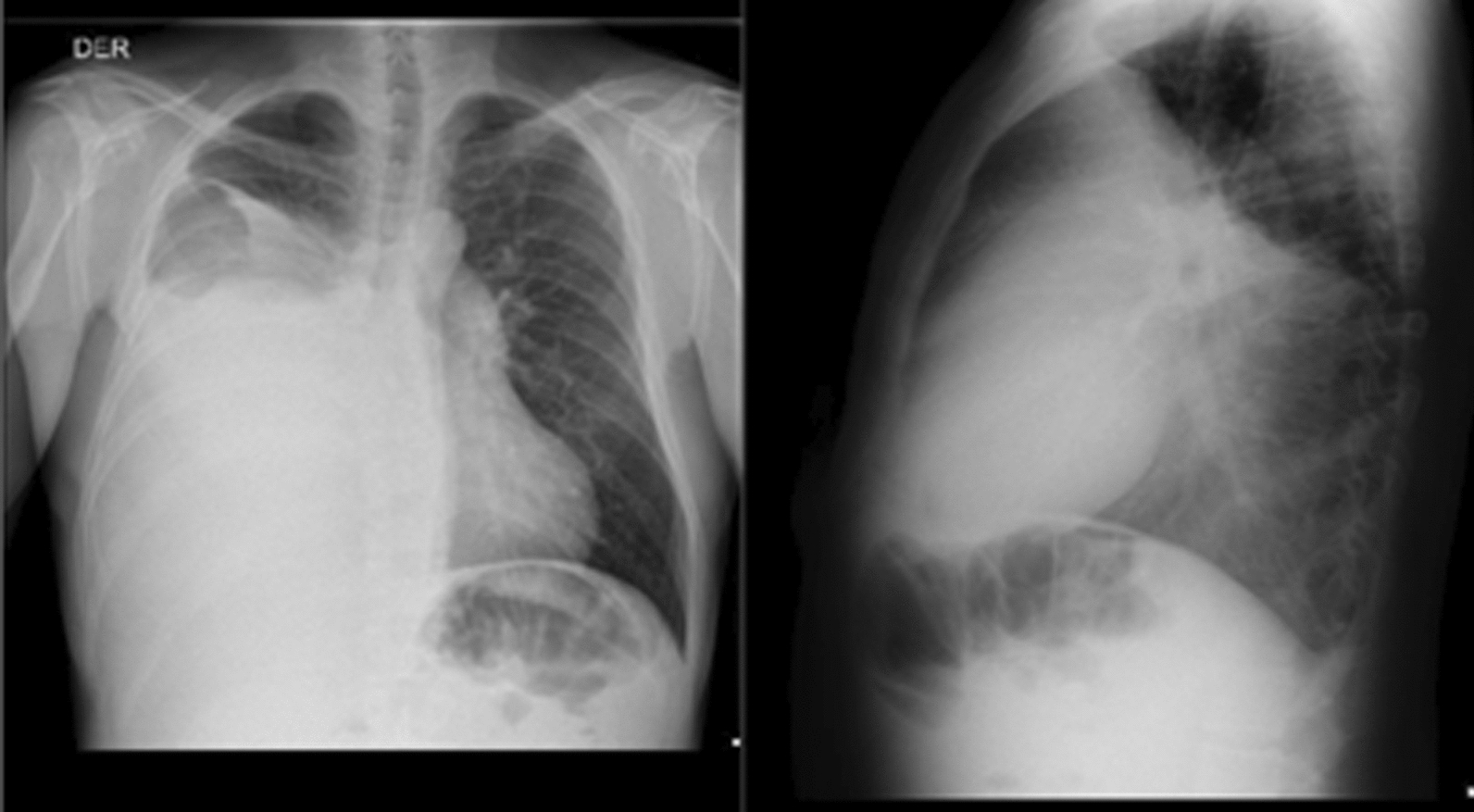
Anteroposterior (AP) and lateral chest radiographs, highlighting a radiodense space-occupying lesion with ill-defined borders and mild ipsilateral pleural effusion, accompanied by subsegmental atelectasis in the ipsilateral hilar regions

**Fig. 3:**
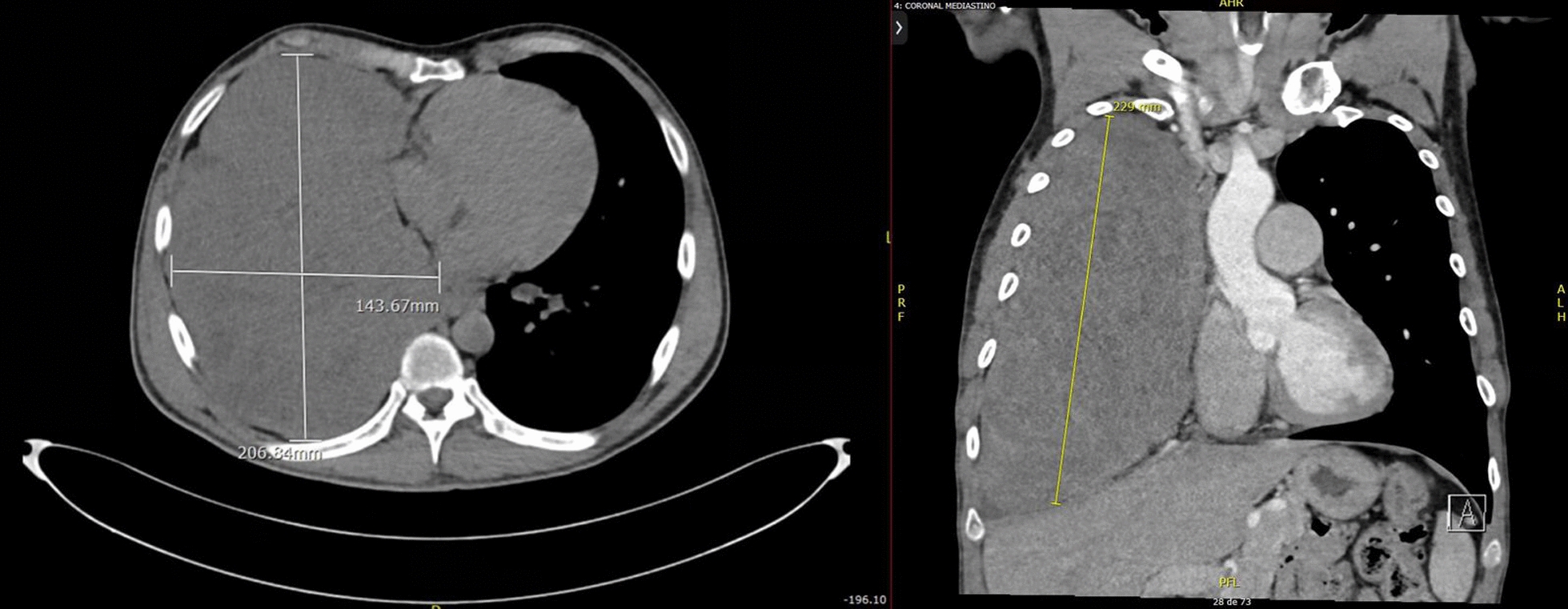
Chest computerized tomography (CT) of axial (left) and coronal (right) sections revealed a large solid mass with extrapulmonary features, demonstrating mild heterogeneous enhancement and some coarse calcifications in its lower portion, measuring approximately 229 by 204 by 124 mm, occupying nearly the entire right hemithorax. An apparent vascular supply was observed from branches of the right middle adrenal artery and right internal mammary artery

Mixed delirium was considered the initial working diagnosis. The admission laboratory results are presented in Table 1. Neurologic services ruled out structural central nervous system components through cranial CT and contrast-enhanced cerebral magnetic resonance imaging (MRI), both of which were normal. Twelve-hour electroencephalography showed no ictal activity. According to the described history and semiological findings, a chest X-ray (Fig. 2) revealed a radiopaque mass occupying the right hemithorax. Thoracic surgeons indicated that an incisional biopsy after a chest CT scan (Fig. 3) revealed a pleura-dependent mass affecting the right hemithorax and compressing the ipsilateral lung parenchyma.

**Table 1:** Case laboratory parameters

Laboratories	Result	Unit of measure	Reference range
*Blood count*			
Leukocytes	7.4	10^3^ Cel/Ul	5.0–10.0
Neutrophils	4.8	10^3^ Cel/Ul	2.0–7.0
Lymphocytes	1.7	10^3^ Cel/Ul	1.5–4.0
Hemoglobin	16.7	g/dl	13.5–18
Hematocrit	50.7	%	40–54
Platelets	258	10^3^ Cel/Ul	150–450
*Electrolytes*			
Sodium	140	mEq/l	136–142
Potassium	3.9	mEq/l	3.5–5.5
Calcium	8.4	mg/dl	8.4–10.2
Chloride		mEq/l	98–107
Magnesium	1.61	mg/dl	1.6–2.6
*Renal function*			
Creatinine	0.6	mg/dl	0.6–1.1
Urea Nitrogen	8	mg/dl	6–20
*Hepatic function*			
Total Bilirubin	1.9	mg/dl	0.2–1.5
Direct Bilirubin	0.6	mg/dl	0–0.5
Aspartate Transaminase	13	U/l	5–34
Alanine Transaminase	14	U/l	0–55
Alkaline Phosphatase	49	U/l	40–150
*Endocrine laboratories*			
C-Peptide	0.06	ng/ml	0.78–5.19
Insulin	< 1.6	U/ml	2.9–19.0
IGF-1	146	ng/ml	94–252
72-h Fasting Test			
Non-hyperinsulinemic Hypoglycemia			
HbA1c	5.10%	%	< 5.7
TSH	1.97	mUI/l	0.35–4.94

During the procedure, the patient experienced a relapse of neurological symptoms, including stupor, upwards gaze deviation, and profuse diaphoresis, which were identical to what family members had reported. A glucometer reading of 35 mg/dl indicated severe symptomatic hypoglycemia. Intravenous correction with 10% dextrose in distilled water resulted in full symptom reversal, recovery of consciousness with amnesia of the incident, and further biochemical investigations (Table 1).

**Fig. 4:**
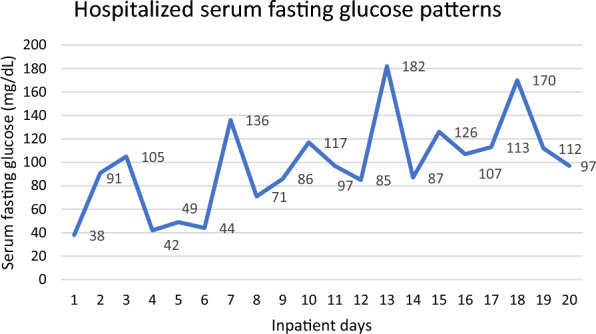
Hospitalized serum fasting glucose patterns

Despite a constant intravenous 10% dextrose infusion rate of 20 cc/h, the patient continued to experience episodes of hypoglycemia. The addition of 10 mg of prednisolone resulted in the remission of neuroglycopenic episodes and glycemic control (Fig. 4).

**Fig. 5:**
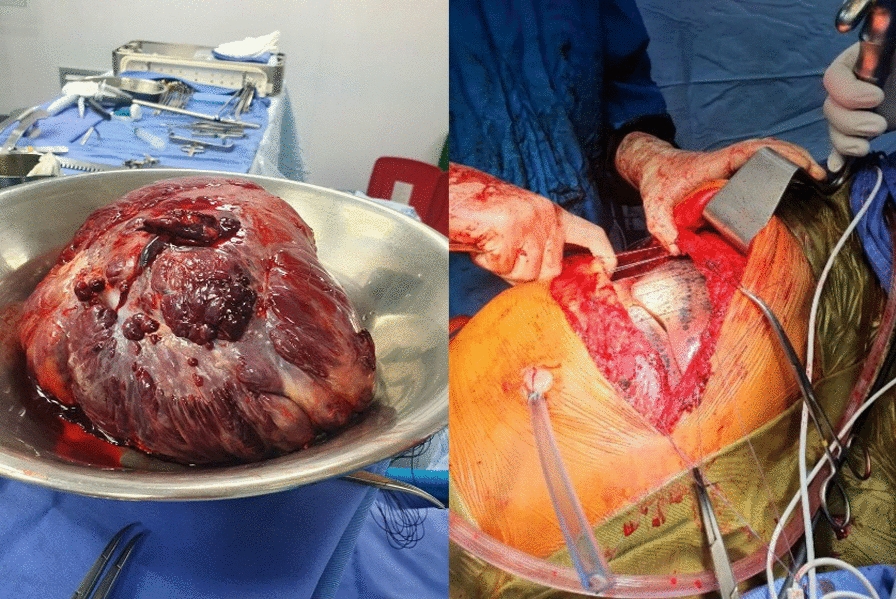
(Left) Large right intrathoracic mass measuring 25 by 18 by 20 cm, originating from the medial and subpulmonary aspects of the right lower lobe, with multiple adhesions. (Right) Complete lung expansion after mass removal without lung compromise

**Fig. 6:**
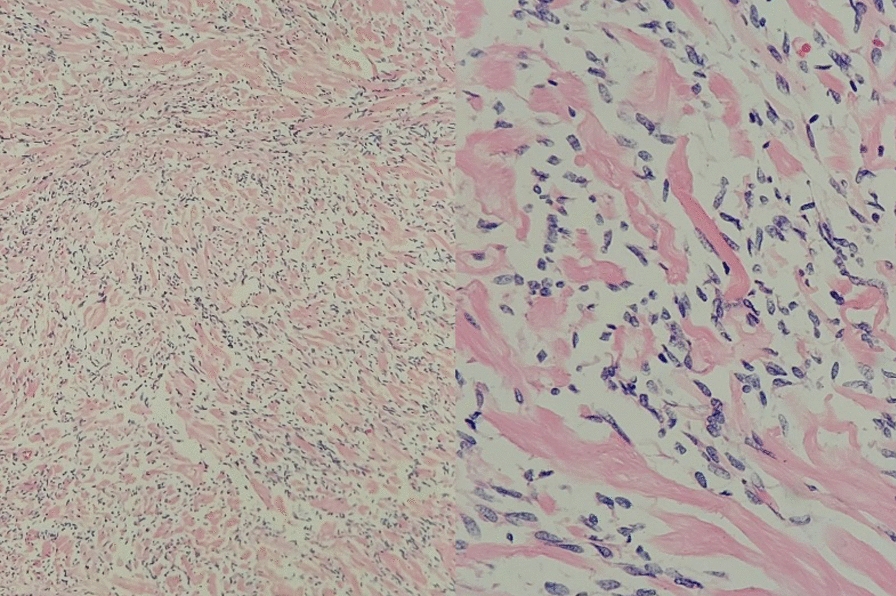
Fusocellular tumors with moderately dense collagenous stroma, delicate and branching vasculature, devoid of mitotic activity, necrosis, cellular atypia, or areas of hypercellularity. Low-risk solitary fibrous tumor stratification was performed according to the 2017 DEMICCO et al. [17] criteria

An open approach involving right thoracotomy was used for tumor removal, and paraneoplastic phenomena due to the intrathoracic mass were considered. Nonanatomic lung resection of the anterobasal segment of the right lower lobe was used to remove the entire tumor. The infiltrative involvement and secondary adhesions of the tumor pose difficulties for surgical treatment, especially when releasing the vascular pedicle. In the end, a total resection of the 25 × 18 × 20 cm mass was obtained (Fig. 5). A solitary fibrous tumor of the pleura was confirmed by histopathological investigation (Fig. 6) and classified as low risk by modified risk stratification criteria proposed by DEMICCO et al. in 2017 [17].

**Fig. 7:**
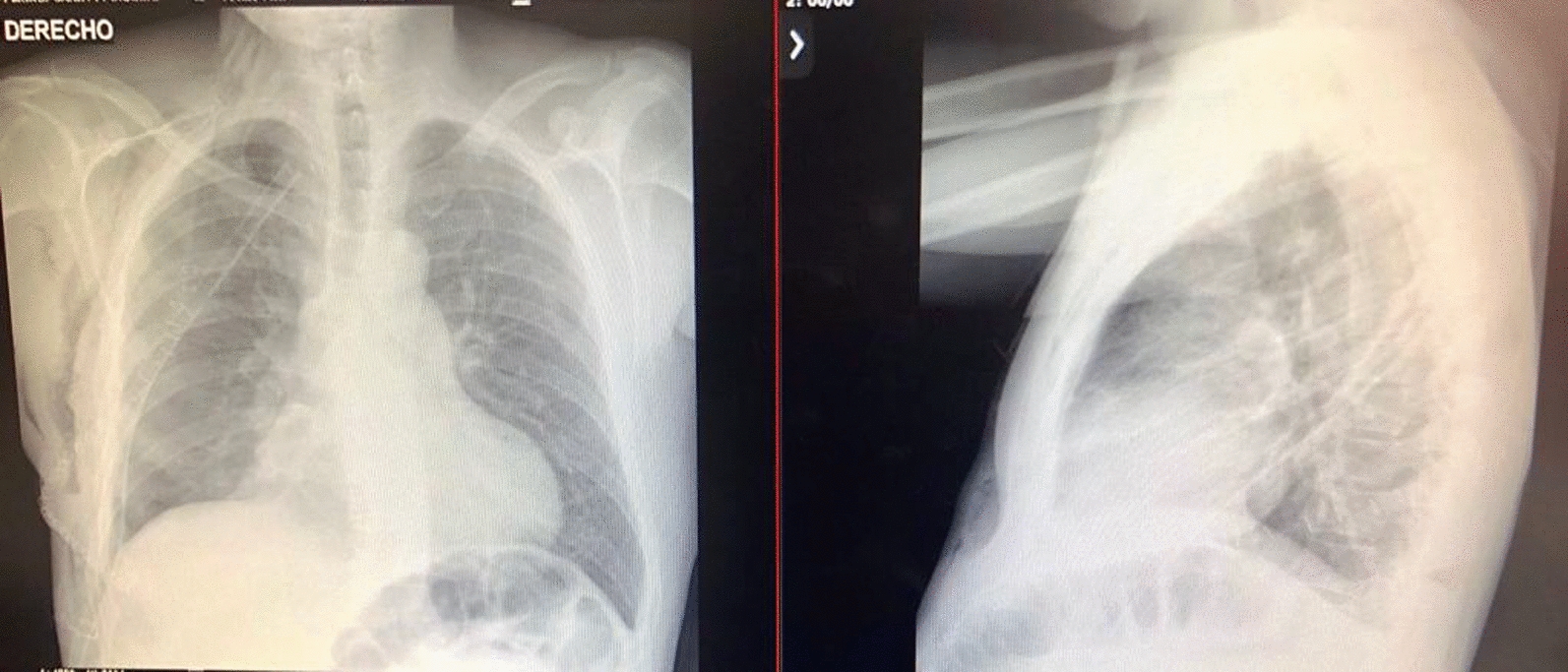
Postoperative anteroposterior (AP) and lateral chest radiography demonstrating lung re-expansion, with clear costophrenic and cardiophrenic angles

**Fig. 8:**
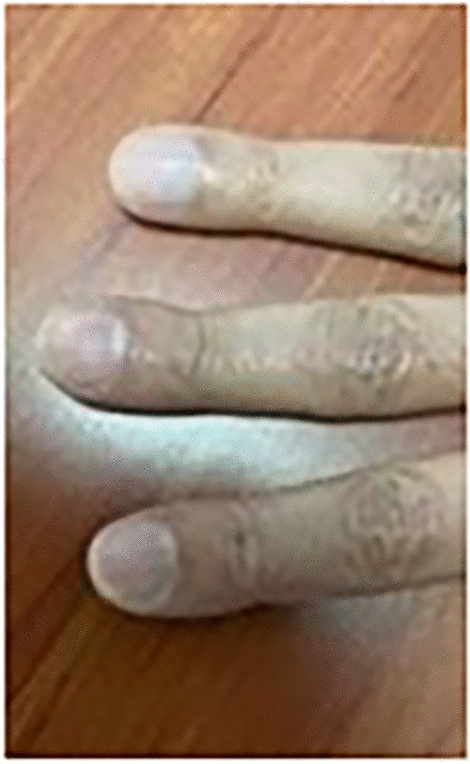
Image of the patient during the ambulatory follow-up period, revealing evidence of resolution of cutaneous changes

Adequate lung re-expansion was observed on postoperative chest X-ray (Fig. 7). The patient maintained good glycemic control during the postoperative period. Consequently, he was discharged after 5 days with a nasal cannula for oxygen after five days, along with orders for follow-up visits with Thoracic Surgery, Endocrinology, and Pulmonology. The patient had a Follow-up check up at3, 6, and 12 months post-discharge, during which blood glucose levels were evaluated and found to be within normal range. Additionally, there was an improvement in digital clubbing (Fig. 8), as evidenced by the previously described images. The patient was still free of tumor recurrence.

## Discussion

A classic instance of Doege-Potter syndrome was documented, with a solitary fibrous tumor of the pleura causing difficult-to-treat severe symptomatic hypoglycemia. Despite having all neuroglycopenic (behavioral changes, drowsiness, confusion), adrenergic (anxiety, tremor, palpitations), and autonomic (hunger, diaphoresis) symptoms, hypoglycemia is rarely diagnosed in non-diabetic patients [15]. And if it is suspected, generally it is considered to have more common causes outside of insulin, like drug side effects (sulfonylureas, antibiotics), alcohol, malnutrition, liver or kidney failure, and endocrine causes (endogenous hyperinsulinism and adrenal insufficiency) [15]. As a result, non-pancreatic malignancies are seldom considered to cause hypoglycemia, yet it can be the only manifestation of the disease [12].

Solitary fibrous tumors (SFTs) are rare soft tissue neoplasms that develop from submesothelial mesenchymal cells with fibroblastic differentiation [18]. Although first described as intrathoracic, the majority of instances come from the visceral pleura, accounting for less than 5% of all pleural malignancies. They may additionally arise within the lungs from the parenchyma, mediastinum, or diaphragm. Other common tumor sites are the retroperitoneum, pelvis, liver, and mediastinum [18, 19]. They are typically benign tumors with slow growth that become symptomatic only when they reach a significant size, resulting in cough, dyspnea, chest discomfort, pleural effusion, or digital clubbing [16], as shown in our instance.

Hypoglycemia associated with these tumors is infrequent and is known as Doege-Potter syndrome, accounting for less than 5% of all cases [5, 20]. In this condition, changes in IGF-2 folding result in the generation of bigger peptides, which impede cellular receptors signaling pathways of counterregulatory hormones, favoring hypoglycemia states [21]. However, the presence of "large" IGF-2 does not guarantee a relationship with NICTH episodes. Lloyd et al. found that 83.3% of fibrous tumors expressed IGF-2 mRNA, while only 7.1% of patients experienced hypoglycemia [22]. Predictors for the biochemical diagnosis of NICTH include an IGF-2/IGF-1 ratio greater than 3 and inhibition of IGF-1 expression [14, 23]. Although these tests were not done on our patient due to study constraints, other non-insulin-induced hypoglycemia diagnostic criteria, such as reduced insulin levels and undetectable C-peptide, were present [20, 24, 25].

Currently, there are no clinical practice guidelines for NICTH patients. The initial therapy is usually symptomatic, with an intravenous dextrose bolus or infusion while waiting for surgery. In this example, a continuous infusion of dextrose fluids was employed to treat hypoglycemia as an interim measure for tumor removal. Previous research found that corticosteroids at dosages greater than 25 mg/day of prednisolone reduced hypoglycemia episodes by 75% in medically treated individuals by decreasing "large" IGF-2 [26]. Bourciguax et al. demonstrated the efficacy of combination corticosteroid and growth hormone therapy in treating hypoglycemic episodes [27]. The use of glucagon is more controversial, with inconsistent outcomes [14].

The definitive treatment is a full surgical excision of the tumor with clear margins [18, 25]. The histological report of the neoplasia has no bearing on the curative surgical technique. Tumor size determines the type of surgery performed: thoracotomy for large tumors orthoracoscopy for small tumors. Complete mass excision has a 5-year survival rate of 100%, with a recurrence risk of 20% [28]. In our case, nonanatomic excision of the anterobasal portion of the lower right lobe was done by thoracotomy. Vascular pedicle inscision was made for total tumor mass release and subsequent linear mechanical suturing. Adequate postprocedural lung re-expansion was demonstrated, indicating the likelihood of total excision (Fig. 2). Other therapeutic options for cytoreduction, such as glucocorticoids, chemotherapy, and radiotherapy, were not investigated in this case, as surgery was provided as a curative attempt [25]. Anthracycline-based chemotherapy regimens [29] are available for individuals whose cancers are unresectable or have metastatic disease. In other cases, combination therapy with temozolomide and bevacizumab may be recommended [20]. There is currently insufficient data to support the use of radiotherapy, and there are few descriptive studies and case reports using brachytherapy and photodynamic therapy during the preoperative period [25].

When compared to other recorded Doege-Potter syndrome cases around the world and in Colombia, some epidemiological points stand out. The patient's age at clinical presentation was lower than that documented in the literature, with the disease peaking between the sixth and eighth decades of life [10]. Furthermore, this is the first recorded male incidence in Colombia in contrast to two prior cases in females [15, 16], for a syndrome in which it is uncertain if women are more affected than men [9]. Additionally, it is a case of non-insulin-induced hypoglycemia (NICTH) caused by a benign tumor, which is generally associated with malignancies in up to 56% of situations [14]. Finally, a right-sided pleural tumor with a right-to-left ratio larger than 2 was found, which is indicative of a tumor linked to hypoglycemia [14].

Regarding the strengths and limitations of our approach, it is worth noting that the previous medical record of lung mass skewed the initial approach, which aimed to rule out intracerebral metastases while overlooking metabolic causes on admission analytics. Yet, once hypoglycemia was identified, interdisciplinary collaboration across internal medicine, thoracic surgery, and endocrinology allowed recognition and medical attention of Doege-Potter syndrome, highlighting the value of teamwork. We acknowledge that the measurement of IGF-2 was not performed, and this laboratory result could have supported the diagnosis and served as a prognostic biomarker to define the risk of recurrence in light of the available literature. Aside from that, we believe our experience will be extremely beneficial to other healthcare professionals by raising awareness of Doege-Potter syndrome, enhancing early detection and effective patient management, and motivating future clinical research for evidence-based treatment guidelines.

## Conclusions

As previously stated, NICTH is a difficult metabolic illness characterized by aggressive disease behavior, local recurrence, and metastatic involvement [26]. Counterregulatory hormones are frequently used to offset the endogenous activity of paraneoplastic hormones, but there is limited evidence. The relevance of prognostic biochemical, histological, and imaging parameters, such as IGF-2 levels, vimentin, CD34, bcl-2, or CD99 positivity but cytokeratin negativity, and tumor size > 10 cm, is unclear [13]. Raising knowledge among healthcare providers about Doege-Potter syndrome patients may serve to improve diagnostic suspicion, biochemical confirmation, and the creation of diagnostic and treatment guidelines. Future advancements are expected in developing models that can forecast aggressive disease presentations that necessitate closer monitoring.

## Data Availability

All data generated and analysed during the current study are included in this published article [and its supplementary information files].

## Abbreviations

CPAP: Continuous positive air pressure
CT: Computed tomography
IGF: Insulin-like growth factor
MRI: Magnetic resonance imaging
NICTH: Nonislet cell tumor hypoglycemia
SFTP: Solitary fibrous tumors of the pleura
SFTs: Solitary fibrous tumors
